# Lydicamycins induce morphological differentiation in actinobacterial interactions

**DOI:** 10.1128/aem.00295-25

**Published:** 2025-05-13

**Authors:** Scott A. Jarmusch, Morten D. Schostag, Zhijie Yang, Jinglin Wang, Aaron J. C. Andersen, Tilmann Weber, Ling Ding

**Affiliations:** 1 Department of Biotechnology and Biomedicine, Technical University of Denmark 158981 , Kongens Lyngby, Denmark; 2 The Novo Nordisk Foundation Center for Biosustainability, Technical University of Denmark 587234, Kongens Lyngby, Denmark; University of Delaware, Lewes, Delaware, USA

**Keywords:** *Streptomyces*, microbial interactions, mass spectrometry imaging, multi-omics, actinobacteria

## Abstract

*Streptomyces* are major players in soil microbiomes; however, their interactions with other actinobacteria remain largely unexplored. Given the complex developmental cycle of actinobacteria, a multi-omics approach is essential to unravel the interactions. This study originated from the observation of induced morphogenesis between two environmental isolates from the same site, *Kitasatospora* sp. P9-2B1 and *Streptomyces* sp. P9-2B2. When co-cultivated on potato dextrose agar, P9-2B2 triggered a wave-like sporulation pattern in strain P9-2B1. Mass *s*pectrometry imaging revealed that a suite of lydicamycins accumulated in the induced sporulation zone. Using CRISPR base editing, lydicamycin-deficient mutants were generated, and the inducible sporulation was ceased, confirming the role of lydicamycin in triggering morphological differentiation. In agar diffusion assays, pure lydicamycin was inhibitory when added concurrently with bacterial inoculation but induced sporulation when added later. The same inducible sporulation wave phenomenon was also observed in additional environmental isolates and *Streptomyces coelicolor* M145 and M1146. Transcriptomics analysis revealed differential gene expression linked to early aerial mycelium development at 4 days into co-culture, the transitional genes responsible for the development of spores at day 9, together with numerous genes for overall stress responses, particularly cell envelope stress responses. These findings highlight previously unrecognized actinobacteria interactions mediated by lydicamycins, suggesting a broader ecological role of bioactive metabolites in microbiomes.

**IMPORTANCE:**

Moving beyond an antibiotic discovery mindset, exploring the chemical ecology of secondary metabolites is key to maximizing their biotechnological potential. Dual cultures offer reduced complexity, enabling an in-depth analysis of these interactions via multi-omics, which provides complementary data for more robust conclusions. This study sheds light on the role of lydicamycins in dual cultures with other actinobacteria and establishes an integral roadmap for future chemical ecology work between microorganisms, particularly through mass spectrometry imaging.

## INTRODUCTION

*Streptomyces* are one of the most widely utilized natural sources, principally for their prolific production of ~70% of clinical antibiotics (1, 2). The attention to this veritable “arms race” has diverted resources toward drug discovery, whereas in contrast, the wider ecological influence of *Streptomyces* secondary metabolites (SMs) has severely lagged behind, despite few reported signaling molecules, for example, γ-butyrolactones (3), governing the production of other SMs or transition of life stage. *Streptomyces* serve vital roles in soil microbiomes, evidenced by their diverse ecological distribution, and their SMs are the “language” they communicate with friends and foes (4, 5). Although interesting observations have been found for microbial inter- and intra-kingdom interactions, such as *Streptomyces*-fungus (6) and *Streptomyces-Bacillus subtilis* interactions (7), respectively, a large gap exists in understanding the role SMs play in actinobacteria interactions.

Sporulation is a common phenotype change observed in *Streptomyces* interactions (8); however, tracing the responsible SMs has rarely been studied. Goadsporin was the first SM described to cause sporulation in *Streptomyces* (9). A major challenge is linking the phenotype change to the secondary metabolite; identifying the metabolite from the earliest stages of analysis is the bottleneck allowing for efficient linkage of SMs and ecology. Mass spectrometry imaging (MSI) offers attractive solutions to resolving this spatial problem (10, 11) through direct visualization of metabolites on co-cultures (12). In *Streptomyces*-actinobacteria studies, Traxler et al. set the primer for evaluating actinobacterial co-cultures, leading to the discovery of new acylated desferrioxamines (13), and many studies have followed, thereafter proving that MSI on co-cultures is a faster route to the discovery of novel compounds.

During a screening for *Streptomyces*-actinobacteria antibiosis, one environmental strain (*Streptomyces* sp. P9-2B2) induced a wave of sporulation in a receiver strain (*Kitasatospora* sp. P9-2B1). Using a combination of MSI, molecular genetics, and biological testing, we determined that P9-2B2 produced a suite of antibiotic lydicamycins responsible for the induced morphogenesis. The temporal production of lydicamycins was evaluated using liquid chromatography–tandem mass spectrometry (LC-MS/MS) and feature-based molecular networking. Finally, we performed transcriptomics analysis on a coculture between *Streptomyces coelicolor* M1146 and P9-2B2 to determine the differentially expressed genes during lydicamycin exposure.

## MATERIALS AND METHODS

### Selective isolation and genomics

All environmental isolates used in this study were isolated from soil collected in 2020 from the UNESCO World Heritage Site, Jægersborg Deer Park (Dyrehaven), Denmark. Selective isolation using standard techniques was carried out to isolate actinobacteria and specifically Streptomycetes (14, 15). The genome sequence data were analyzed with the Type Strain Genome Server (TYGS), a free bioinformatics platform available under https://tygs.dsmz.de, for a whole genome-based taxonomic analysis. Both P9-2B1 and P9-2B3 were identified as isolates of *Kitasatospora papulosa* (digital DNA-DNA hybridization, dDDH, of 94.2% and 93.4% compared with *Kitasatospora papulosa* NRRL B-16504), whereas P9-2B2 and P9-2B4 appeared as *Streptomyces platensis* (dDDH of 74.1% and 76.2% compared with *Streptomyces platensis* DSM40041). More detailed methods and phylogenetic trees can be found in the supplemental material (Fig. S1 to S4). The genome sequences of *Kitasatospora* sp. P9-2B1 and *Streptomyces* sp. P9-2B2 were deposited into BioProject PRJNA985726. *S. coelicolor* M145 and M1146 were obtained from Prof. Mervyn Bibb, John Innes Centre, Norwich.

### Actinobacterial co-cultures and monocultures

All spore stocks used in this study were standardized to 10^5^ CFU/mL, 10 µL of inoculum was added for each isolate, and all co-cultures were carried out on BD Difco Potato Dextrose Agar (PDA). All co-cultures were spaced 1.5 cm apart. Inoculant used for MSI was incubated at 30°C for 7 days and then prepared for imaging. Inoculate used for timelapse imaging were placed in a 30°C incubator room with a Reshape Timelapse Imager (Reshape, Copenhagen, DK), and one image was taken every 60 min for 7–10 days, depending on experiment length. Experiments involving pure lydicamycin (Santa Cruz Biotechnology Inc.) bacterial spore stocks were plated and pre-incubated for 3 days, and then, pure metabolites were added to an agar well 1.5 cm away. Pre-incubation was carried out to (i) mimic the time point lydicamycin production is seen in P9-2B2 monocultures and (ii) prevent inhibitory effects of the metabolite. P9-2B2 monocultures for temporal SM production: 10 PDA plates with three 10 µL spots were incubated, where every day one plate was removed, and one colony was imaged using a Leica S9i digital microscope and extracted as described below. The exact same procedure was carried out with ISP2 plates but only sampling on days 1, 2, 3, 4, 7, and 10. Supplementary microscopic images were acquired by a Zeiss SteREO Discovery.V12 stereo microscope, equipped with a Zeiss Axiocam 702 mono digital camera (Carls Zeiss AG, Oberkochen, Germany).

### Inactivation of the lydicamycin BGC

In order to inactivate the core biosynthetic gene *lyd60* (NCBI GenBank accession WP_290354452.1)*,* coding for the first modules of the lydicamycin PKS, a STOP codon via CRISPR-BEST base editing (16) (*lyd*60^STOP^) was designed using the online tool CRISPy-web (17). pCRISPR-cBEST plasmid was linearized by NcoI, and NEBuilder HiFi DNA Assembly Master Mix was used to insert the *lyd*60^STOP^ deactivation, resulting in pCRISPR-cBEST/*lyd60*. Subsequently, Mach1 T1 Phage-Resistant Chemically Competent *E. coli* was transformed with the plasmid. The plasmid was re-isolated and transferred into chemically competent *E. coli* ET12567/pUZ8002. The *E. coli-Streptomyces* conjugation experiment was conducted according to the standard protocol (1), and *Streptomyces* mutants were confirmed by Sanger sequencing.

### Sample preparation and mass spectrometry imaging

In total, 10 µL glycerol spore stocks of each microbe were plated onto 4 mm thick PDA agar for all MSI experiments. Sample preparation was adapted from Yang et al. (18). Upon observation of induced sporulation in the receiver strain, colonies were excised using a scalpel and placed on a Bruker IntelliSlide coated with a layer of glue applied with a glue pen. Samples were subsequently dried for 2–4 h at 35°C, followed by matrix application (40 mg/mL DHB, 50:50:0.1% H_2_O:MeOH:TFA) for 15 passes using the HTX-Sprayer (HTX Imaging, Chapel Hill, NC, USA). Samples were further dried at 35°C for 1 h prior to MSI. The samples were then subjected to timsTOF flex (Bruker Daltonik GmbH, Bremen, GE) mass spectrometer for MALDI MSI acquisition in positive MS scan mode with 20 µm raster width and a mass range of 100–2,000 *m/z*. Calibration was done using red phosphorus. The settings in the timsControl were as follows: Laser: imaging 20 µm, Power Boost 3.0%, scan range 16 µm in the XY interval, and laser power 70%; Tune: Funnel 1 RF 300 Vpp, Funnel 2 RF 300 Vpp, Multipole RF 300 Vpp, isCID 0 eV, Deflection Delta 70 V, MALDI plate offset 100 V, quadrupole ion energy 5 eV, quadrupole loss mass 100 *m/z*, collision energy 10 eV, focus pre TOF transfer time 75 µs, and pre-pulse storage 8 µs. All data were analyzed using Bruker SciLS (2021b Pro), and data were normalized to the root mean squared.

### MS-based metabolomics

All extracts for LC-MS were generated using an agar plug method (19). Liquid chromatography was performed on an Agilent Infinity 1290 UHPLC system, and 1 µL extract was injected onto an Agilent Poroshell 120 phenyl-C6 column (2.1 × 150 mm, 1.9 µm) at 40°C using CH_3_CN and H_2_O, both containing 20 mM formic acid. Initially, a linear gradient of 10% CH_3_CN/H_2_O to 100% CH_3_CN over 10 min was employed, followed by isocratic elution of 100% CH_3_CN for 2 min. Then, the gradient was returned to 10% CH_3_CN/H_2_O in 0.1 min, and finally, an isocratic condition of 10% CH_3_CN/ H_2_O for 1.9 min, all at a flow rate of 0.35 min mL^−1^. HRMS data were recorded in positive ionization on an Agilent 6545 QTOF MS equipped with an Agilent Dual Jet Stream electrospray ion (ESI) source with a drying gas temperature of 250°C, drying gas flow of 8 min L^−1^, sheath gas temperature of 300°C, and sheath gas flow of 12 min L^−1^. Capillary voltage was 4,000 V, and the nozzle voltage was set to 500 V. The HRMS data were processed and analyzed using Agilent MassHunter Qualitative Analysis B.07.00. HPLC grade solvents (VWR Chemicals) were used for extractions, whereas LCMS grade solvents (VWR Chemicals) were used for LCMS.

Raw data were converted to .mzML using MSConvert (ProteoWizard) and preprocessed using MZmine 3 (20). Molecular networking was all completed within the GNPS platform (21), which includes Feature Based Molecular Networking (22). Visualization of the molecular networks was completed using Cytoscape 3.8.2. (23).

### RNA extraction and transcriptomic analysis

*Streptomyces* sp. P9-2B2 (producer) was inoculated in the center of the agar plate with *S. coelicolor* M1146 replicates (*n* = 4) surrounding it at a distance of 1.5 cm. The bacteria were grown at 30°C, and at each time point (days 2, 4, 7, and 9), half of the colony facing the center was collected using a 10 µL inoculation loop and transferred to a 2 mL cryovial containing 100 µL phosphate buffer and 200 µL RNAProtect (QIAGEN N.V., Venlo, The Netherlands). Samples were then centrifuged at 8,000 *g* for 5 min, the supernatant was discarded, and samples were snap-frozen in liquid nitrogen and stored in the −80°C freezer, prior to RNA extraction.

RNA extraction was performed using the RNeasy Mini kit following the manufacturer’s instructions, with the addition of bead beading with the FastPrep (MP Biomedicals, Santa Anna, USA) for 30 s at 4500 RPM with 0.1 mm glass beads. DNA was removed with the TurboTM DNase kit (Thermo Fisher Scientific). RNA concentration was estimated with Qubit Thermo Fisher Scientific using the High Sensitivity RNA kit (concentrations ranging from 62 to 781 ng µL^−1^), and RNA integrity was assessed on an Agilent Bioanalyzer 2100 using Agilent RNA 6000 nano kit. All samples had an RNA integrity number (RIN), with an average of 5.5 (range 2,8–9,0). Ribosomal RNA depletion, library preparation, and sequencing were performed at Novogene Co., Ltd on a NovaSeq PE150 platform. A detailed description of RNA-seq analysis can be found in the supplemental methods.

## RESULTS

### Diffusible metabolites induce sporulation of *Kitasatospora* sp. P9-2B1

**Fig 1:**
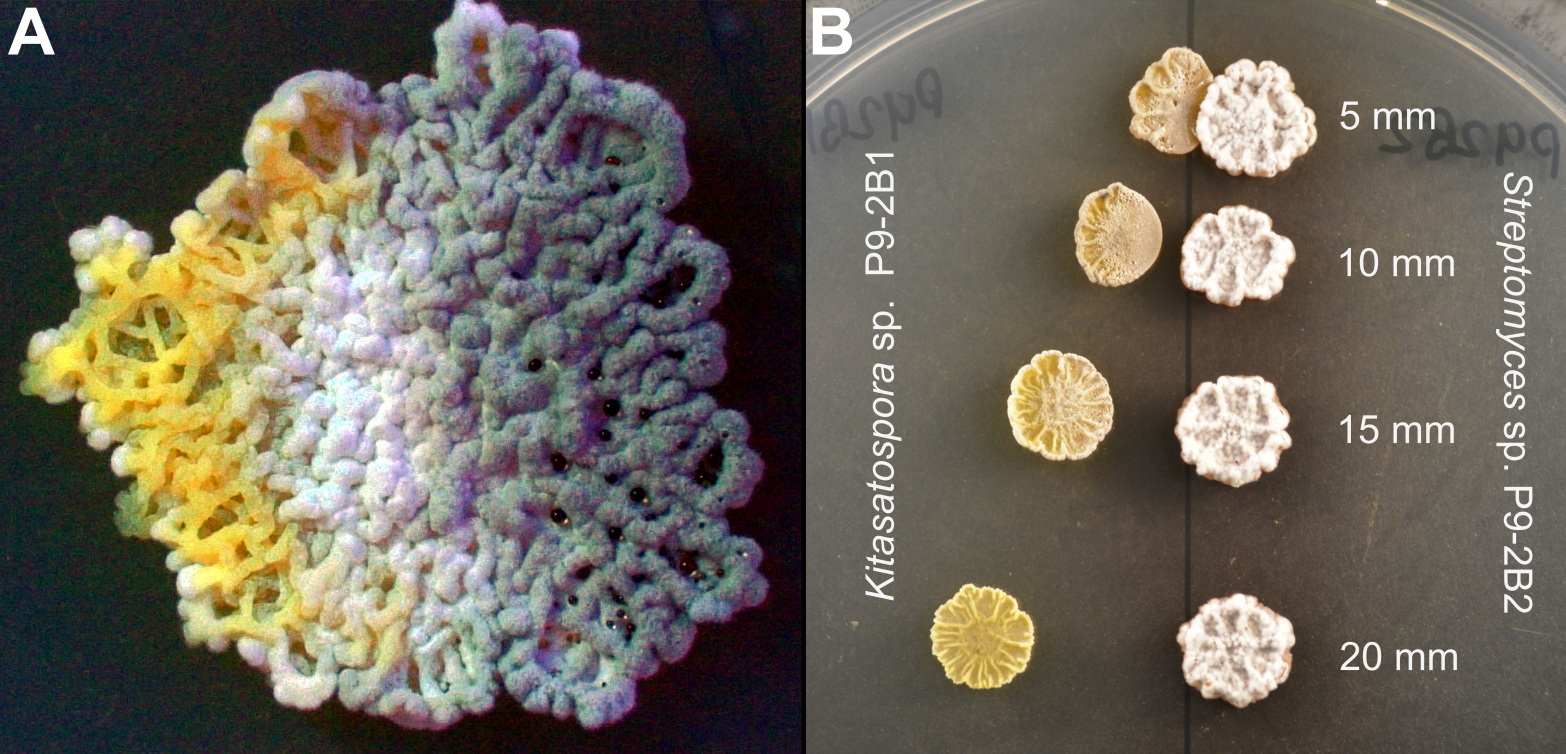
(**A**) Observed differentiation of *Kitasatospora* sp. P9-2B1 on PDA after 8 days in co-culture with *Streptomyces* sp. P9-2B2 (cultured on the right side of P9-2B1). (**B**) Distance-based assay showing the diffusible zone of sporulation induced by secondary metabolite production by *Streptomyces* sp. P9-2B2.

While investigating actinobacteria interactions, pairwise co-culture was carried out, and it revealed that *Streptomyces* sp. P9-2B2 caused sporulation of *Kitasatospora* sp. P9-2B1. After 7 days of growth, P9-2B1 exhibited a zone of sporulation emanating from P9-2B2, which progressed in three distinct phases starting from the edge closest to P9-2B2 (Fig. 1A): (i) substrate hyphae first changed in pigmentation, from pale to bright yellow, (ii) production of aerial hyphae (white), and (iii) production of spores (greenish gray). Although the mechanism behind sporulation and the transcriptional effects of sporulation are well understood, metabolites that cause sporulation are essentially unknown, thus peaking our excitement.

Due to the “wave-like” nature, we proposed that an agar-diffusible metabolite produced by P9-2B2 was causing the phenotype change in P9-2B1. To initially test the hypothesis, a distance-based assay was carried out where P9-2B1 was incrementally (0.5 cm) moved away from the producer, P9-2B2. As P9-2B1 is distanced farther away, the inducible morphogenesis disappears (Fig. 1B). This indicates that diffusible metabolites are indeed responsible for the phenotype change, like previously reported observations of actinobacteria-induced sporulation (8).

### Mass spectrometry imaging illuminates sporulation caused by lydicamycin production

Traditionally, researchers have attempted to characterize chemistry derived from cocultures using standard extraction techniques (12, 19). Therefore, our first attempt to identify the metabolites responsible was via agar plug extracts taken from three distinct locations and analyzed using LC-MS: (i) P9-2B2, P9-2B1 (non-sporulating area), and P9-2B1 (sporulated area). No candidate metabolites were differentially observed when comparing the base peak chromatograms, nor did the chemistry of P9-2B1 change in these sporulated areas (Fig. S5). The lack of identifying any viable SMs with agar plugs and LCMS leads us to use mass spectrometry imaging (MSI) to directly visualize the spatial metabolome.

**Fig 2:**
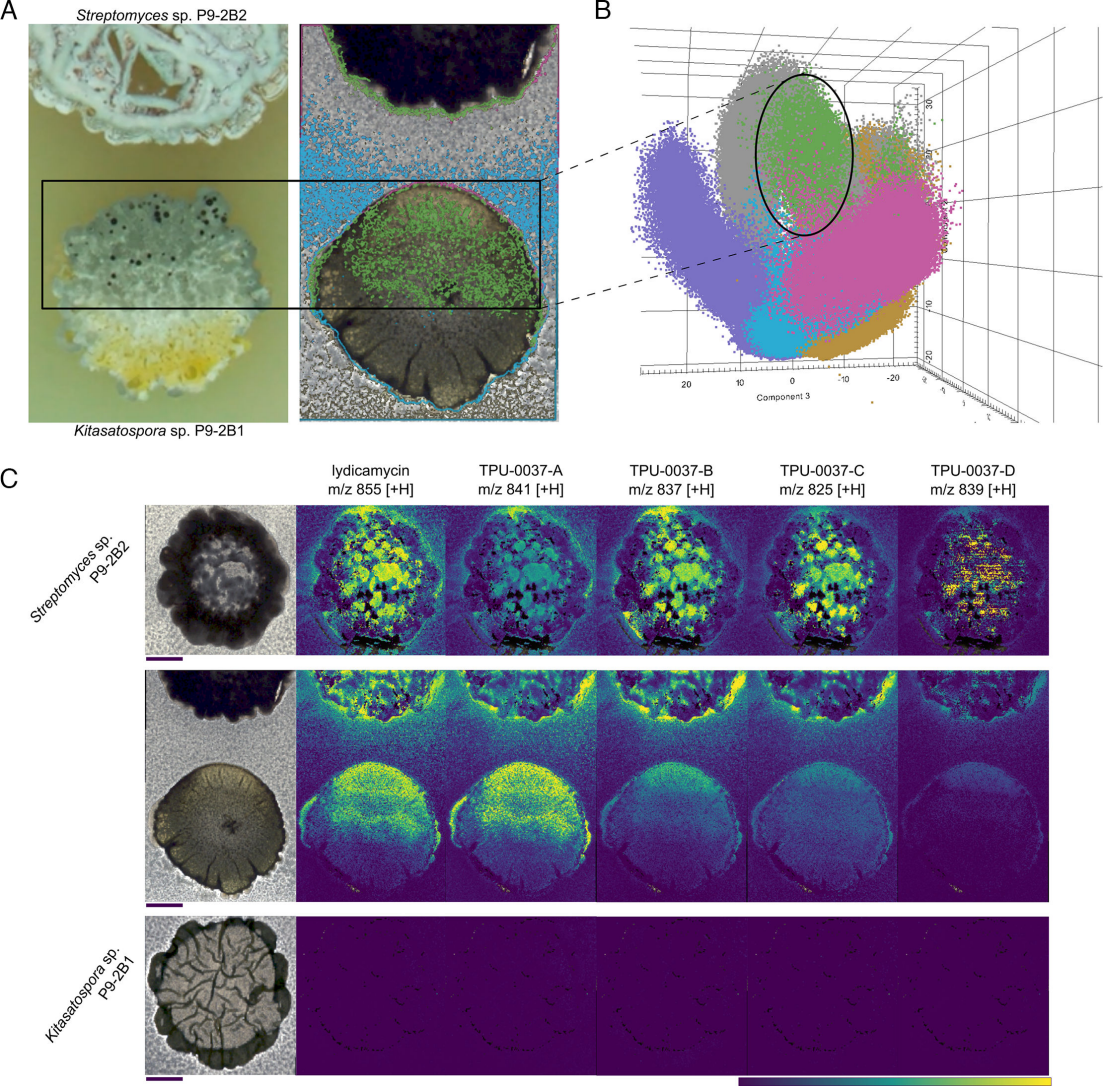
MALDI-MSI of *Kitasatospora* sp. P9-2B1 and *Streptomyces* sp. P9-2B2. (**A**) Digital microscope image of sporulation and interaction zone between the actinobacteria (left) and MSI sampling region with segmentation differentiating P9-2B1 (blue), sporulation zone of P9-2B1 (green), and P9-2B2 (magenta). (**B**) 3D PCA plot of MSI spectra with two additional segments of the P9-2B1 monoculture (lavender) and P9-2B2 monoculture (orange). (**C**) Ion images of the various lydicamycins produced (TPU-0037-A through D are congeners of lydicamycin). Scale bars: 4 mm. Colored scale bar indicates relative ion abundance with hotspot removal activated.

Using an adapted method based on previous studies focusing on imaging microbial interactions on agar (18, 24, 25), actinobacteria interactions were carried out on PDA, excised, and prepared for MSI. The half-sporulated sample of P9-2B1 (Fig. 2A) as well as two monocultures were analyzed using MALDI-MSI. Using Bruker SciLS, we focused on spatially co-localized features ranging from *m/z* 800–860. MSI of the monocultures shows these features are present in and around P9-2B2, the strain causing sporulation. These features were present in the sporulation area of P9-2B1, giving a strong indication that these are the responsible features for the observed sporulation. Furthermore, principal component analysis revealed that the sporulation zone of P9-2B1 (Fig. 2B) was more similar to the surrounding agar and colony of P9-2B2 than to the surrounding agar and non-sporulated colony of P9-2B1. Using BGC predictions from antiSMASH (26) and accurate mass comparison to NPAtlas (27) and MS/MS matching to the GNPS library, we hypothesized the responsible metabolites to be lydicamycins.

**Fig 3:**
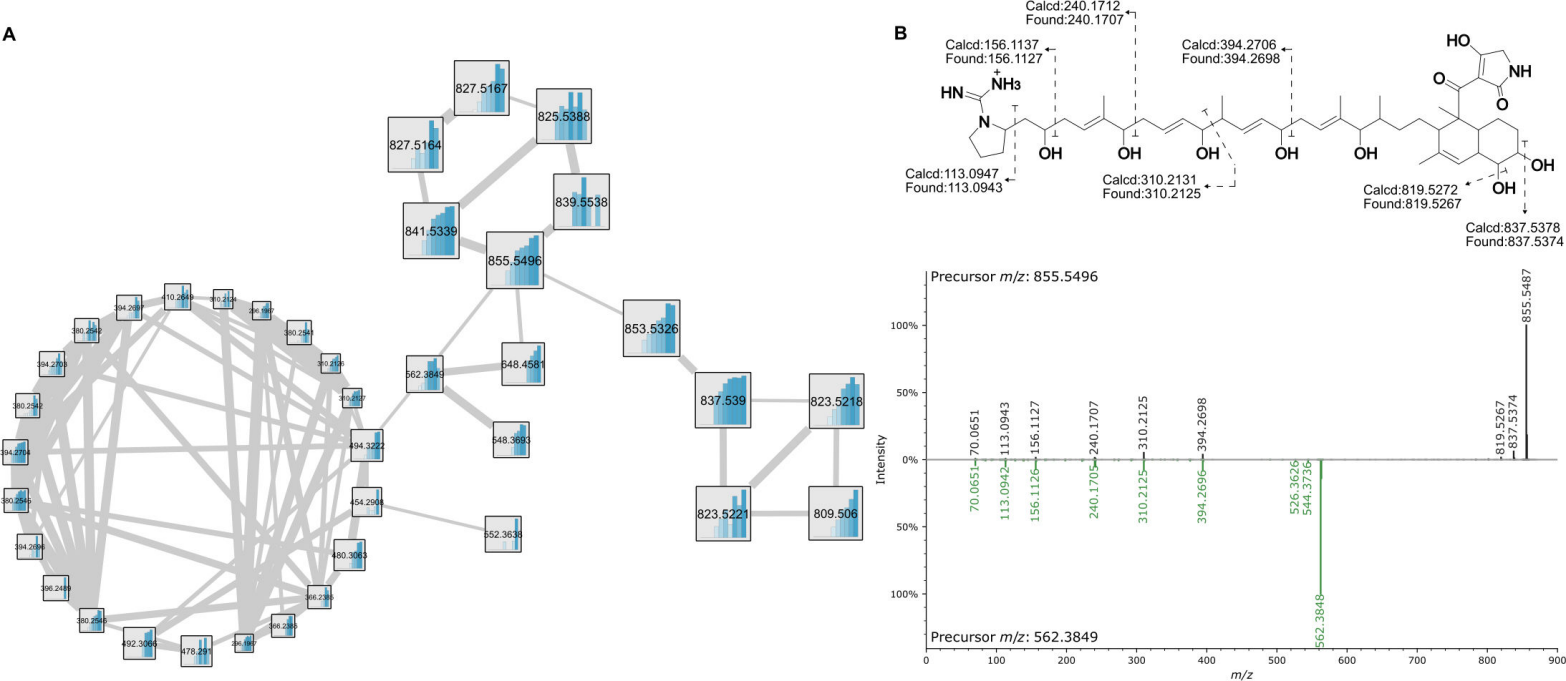
(**A**) Lydicamycin molecular family where increased edge width indicates higher fragmentation similarity. Internal bar graphs indicate peak area over each day from Days 1 to 10. (**B**) Fragmentation of lydicamycin (*m/z* 855.5496) and mirror plot of lydicamycin and the putative shunt product (*m/z* 562.3849). Green highlights indicate a cosine score-based match.

Lydicamycins are non-ribosomal peptide synthetase–polyketide synthase (NRPS-PKS) hybrids, recently named arginoketides (28), that have proposed biosynthesis starting from an enzymatically converted arginine (29) or a separate enzymatic process (30). Five known derivatives were originally described (lydicamycin and TPU-0037-A,B,C,D), and MSI was able to observe all known derivatives (Fig. 2C) (31, 32). On top of the five known lydicamycins detected via MSI, additional lydicamycins were also detected that linked to the known lydicamycins via GNPS molecular networking (Fig. S6). Three features (*m/z* 809.5084 [M + H]^+^, 823.5110 [M + H]^+^, and 853.5326 [M + H]^+^) are structurally related to TPU-0037-B based on molecular networking analysis (Fig. 3A), which contains an additional degree of unsaturation based on accurate mass formula calculations. One feature (*m/z* 827.5167 [M + H]^+^) is structurally related to TPU-0037-A and C. A recent study that also utilized molecular networking to identify new derivatives of lydicamycins also proposed these new congeners which we detect, including *m/z* 811.511 [M + H]^+^, which links in their study to TPU-0037-A (30). They further identify several other features that we also observe (*m/z* 843.5352, 857.5474, 869.53, and 871.54; [M + H]^+^) and additionally, we observe putative analogs (*m/z* 809.5084, 829.5238; [M + H]^+^) which they do not, pointing to the fact there are further products in the biosynthetic pathway to uncover (raw data available). Based on the accurate mass and matching fragmentation spectra to the GNPS library, all confirmed and putative lydicamycins were assigned to Level 2 and Level 3 confidence, respectively, based on Schymanski’s rules (33). All confirmed and putative lydicamycin MS^2^ spectra were deposited into the GNPS Spectral Library (Table S2).

Finally, an additional set of features was connected in the Feature-Based Molecular Networking (FBMN) via lydicamycin (*m/z* 855) yet was smaller in *m/z* (Fig. 3A). Zhang et al. also observed these smaller features in their molecular network yet provided no explanation for their presence (30). Due to their connectivity, we knew their fragmentation was similar, pointing to their structural similarity as well (Fig. 3B). Upon further investigation, the fragments at *m/z* 394.2696, 310.2125, 240.1707, and 156.1126 can all be traced to the polyketide backbone (Fig. 3B) and to the previously reported fragmentation (31), on the C-C bond adjacent to hydroxyls. Due to this fragmentation similarity, we hypothesize that these remaining features are shunt products or byproducts of lydicamycin biosynthesis (34, 35). The study by Deng et al. (35) shows a similar metabolic flux to shunt production as we observe here; upon production of lydicamycins at day 4, we see immediate detection of potential shunt products and an increase toward days 9 and 10. Additional annotations from the GNPS library can be found in Fig. S7.

### Lydicamycin production is strongly tied to aerial mycelium production

**Fig 4:**
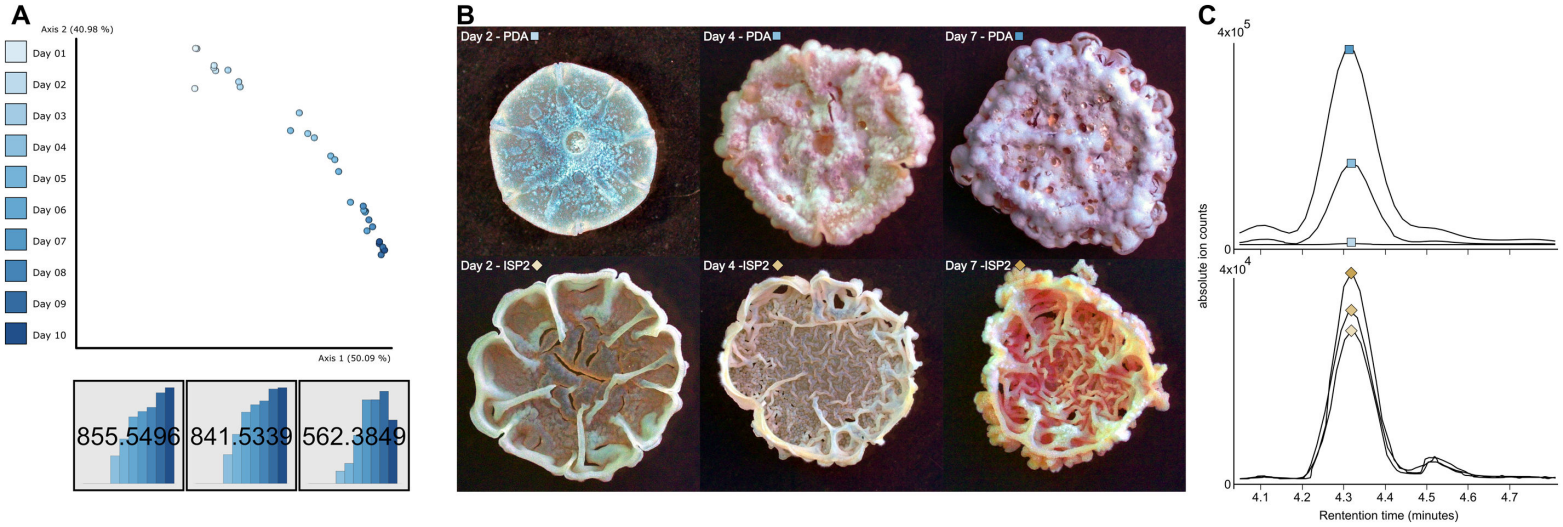
(**A**) PCoA shows the difference in metabolome over the cultivation time of P9-2B2 on PDA. Molecular networking nodes show the production of lydicamycins (*m/z* 855.5496 and 841.5339) and the putative shunt product, *m/z* 562.3849. (**B**) Microscopy of P9-2B2 colonies on PDA and ISP2 from 1, 4, and 7 days of growth on solid agar. (**C**) Extracted ion chromatograms of *m/z* 855.5496 corresponding to the timed images in B for P9-2B2 grown on PDA (top) and ISP2 (bottom).

Due to the role time plays in our coculture system, we sought to investigate when lydicamycins are produced and attempt to link that to morphogenesis. We conducted an untargeted metabolomics analysis over 10 days, where each day a new plate containing three separate macro-colonies was imaged and extracted using plug extraction. Principal coordinate analysis (PCoA) in Qiime2 View (36) allowed us to observe the relative abundances of each feature over this 10-day time scale (Fig. 4A). Furthermore, Pearson r correlation distinguishes three sample groupings: days 1–3, 4–5, and 6–10 (Fig. S8). Nodes from the FBMN (Fig. 4A) indicate all the lydicamycins and the putative shunt products increased in production up to day 9 (saturation of the MS). When comparing these data with the microscopy images taken on each of these days, we observe that morphogenesis and the onset of sporulation strongly correlate with the three metabolomics-based sample groupings: days 1–3 are defined by vegetative growth and no detectable lydicamycin production, days 4–5 are the onset of aerial mycelium and lydicamycin production, and days 6–10 are defined by maturation and sporulation along with the highest level of production (Fig. 4B and C). Supplementary microscopy images can be found in Fig. S9.

In early experiments, we also observed when the two isolates were cocultured on ISP2 agar, neither the receiver strain P9-2B1 had sporulation induced nor did P9-2B2 sporulate itself. Therefore, we hypothesized that lydicamycin production must be lacking due to both observations. The same untargeted time-based experimental setup as described above (with fewer time points) was conducted on ISP2, and the results showed no sporulation in P9-2B2 over 7 days (Fig. 4B). Lydicamycin production began on day 1 when cultured on ISP2 and was stagnant through day 7, but overall, the relative quantities were 10× lower than that on PDA. Based on the timing of lydicamycin production of PDA compared with that of ISP2 and their corresponding phenotypes, we concluded that the onset of P9-2B2 sporulation kickstarts lydicamycin production, a typical phenomenon of secondary metabolite production in *Streptomyces* (37).

### Lydicamycins induce morphogenesis in *S. coelicolor*

Having shown the effect of strain P9-2B2 and lydicamycin production on strain P9-2B1 in this study, we set out to explore if sporulation is also triggered in other environmentally relevant *Streptomyces* from our collection site. Two additional environmental actinobacteria (*Kitasatospora* sp.) P9-2B3 and *Streptomyces* sp. P9-2B4) and *S. coelicolor* M145 and M1146 were cocultured with P9-2B2, and timelapse images for each were taken. The results showed that *Kitasatospora* sp. P9-2B3 and the two *S*. *coelicolor* isolates exhibited the same phenotype change as P9-2B1 (Fig. S10). Sporulation waves began to appear for P9-2B3 starting on day 7 and progressing toward full sporulation on day 10 (Fig. S10), similar to P9-2B1. Based on phylogenetic tree reconstruction, strains P9-2B1 and P9-2B3 are both highly similar to each other, and likewise for P9-2B2 and P9-2B4 (Fig. S1-4). Timelapse videos of each interaction can be found in the supplemental material (Video S1–S4).

Sporulation of *S. coelicolor* M145 has been shown to be inhibited by prodiginine (38), which could explain why we observed delayed sporulation on day 10 and did not fully sporulate by day 21. Strong prodiginine production could be seen starting on day 4, which has been implicated in delaying sporulation in *S. coelicolor* (38), and actinorhodin production was observed in different sporulating regions across days 10–21 (Video S5). Therefore, to remove the effect of the prodiginines, we carried out subsequent work on *S. coelicolor* M1146, which contains four deleted BGCs, including prodiginines (39). In contrast to M145, sporulation in M1146 occurred after 4 days, with full sporulation seen 24 h later (Video S6).

**Fig 5:**
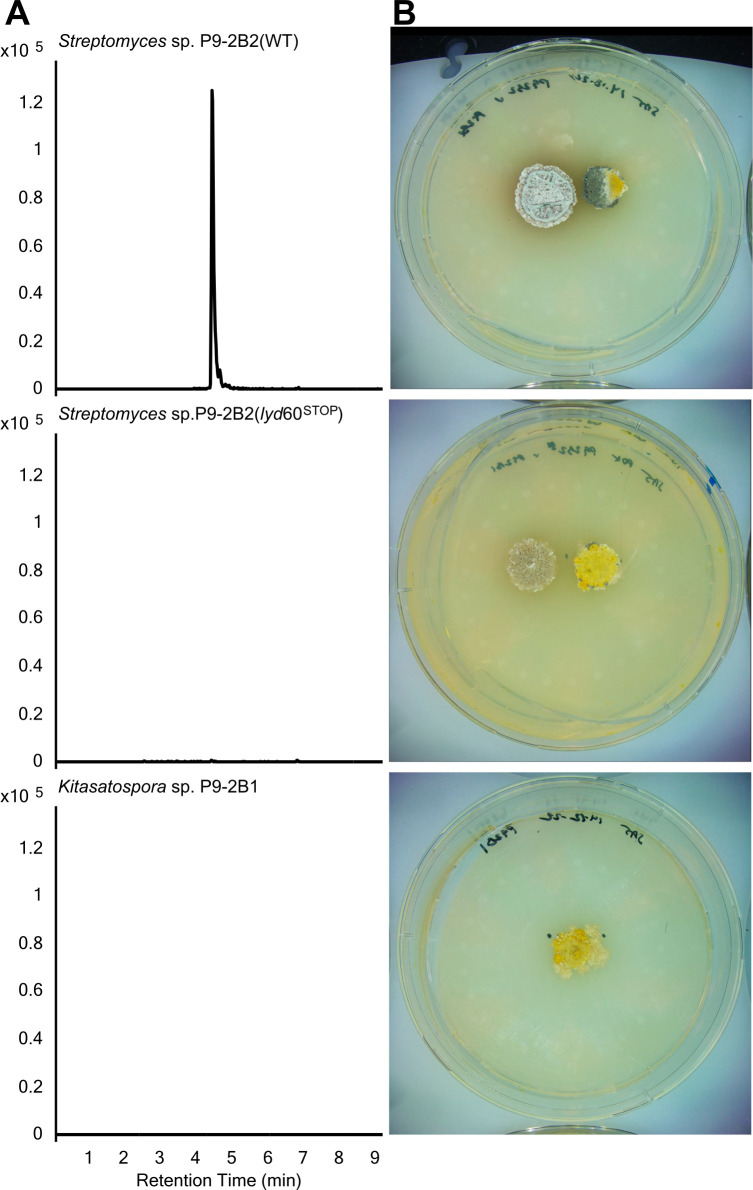
(**A**) Extracted ion chromatogram of lydicamycin (*m/z* 855.5496) in co-culture of WT P9-2B2 and P9-2B1 (top), co-culture of *lyd*60*^STOP^* and P9-2B1 (middle), and monoculture of P9-2B1 (bottom). (**B**) Corresponding images from each co-culture where P9-2B2 is present on the left and P9-2B1 on the right.

To confirm our lydicamycin inducible-sporulation hypothesis based on imaging, metabolomics, and genomics, we generated a *lyd*-deficient mutant to test in coculture. The first core polyketide synthase gene *lyd60* was targeted and inactivated by converting a TGG (Trp) codon at position 57 into the stop codon TAA using the CRISPR-based base-editing tool CRISPR-BEST (16). The deficient mutant (*lyd*60^STOP^) was confirmed by Sanger sequencing of the editing site, with no detectable lydicamycin after 7 days of growth on PDA using LC-MS (Fig. S11), and with co-cultures showing a lack of induced morphogenesis in *Kitasatospora* sp. P9-2B1 (Fig. 5). Subsequent timelapse images were taken over 10 days, and no sporulation was observed in the receiver strain, P9-2B1 (Video S8). *lyd6*0^STOP^ had observably different growth in the first 2 days from the wild type (WT). The first indication of aerial mycelium in WT P9-2B2 appears on day 1 hour 13 (Video S7) compared with day 4 for the *lyd*-deficient mutant (Video S8), indicating the inactivation of the *lyd* BGC delays development in P9-2B2. To further confirm lydicamycin’s role in inducible morphogenesis, pure lydicamycin (*m/z* 855) was added into an agar well and induced sporulation in P9-2B1 but only at 100 µg and a longer interval (9 days) than when cocultured (6–7 days). Sporulation assays were conducted on pregrown cultures for 3 days; otherwise, P9-2B1 was inhibited by pure lydicamycin when cultured simultaneously, indicating its overall antimicrobial effect.

### Genes controlling aerial mycelium are expressed upon increased lydicamycin exposure

**Fig 6:**
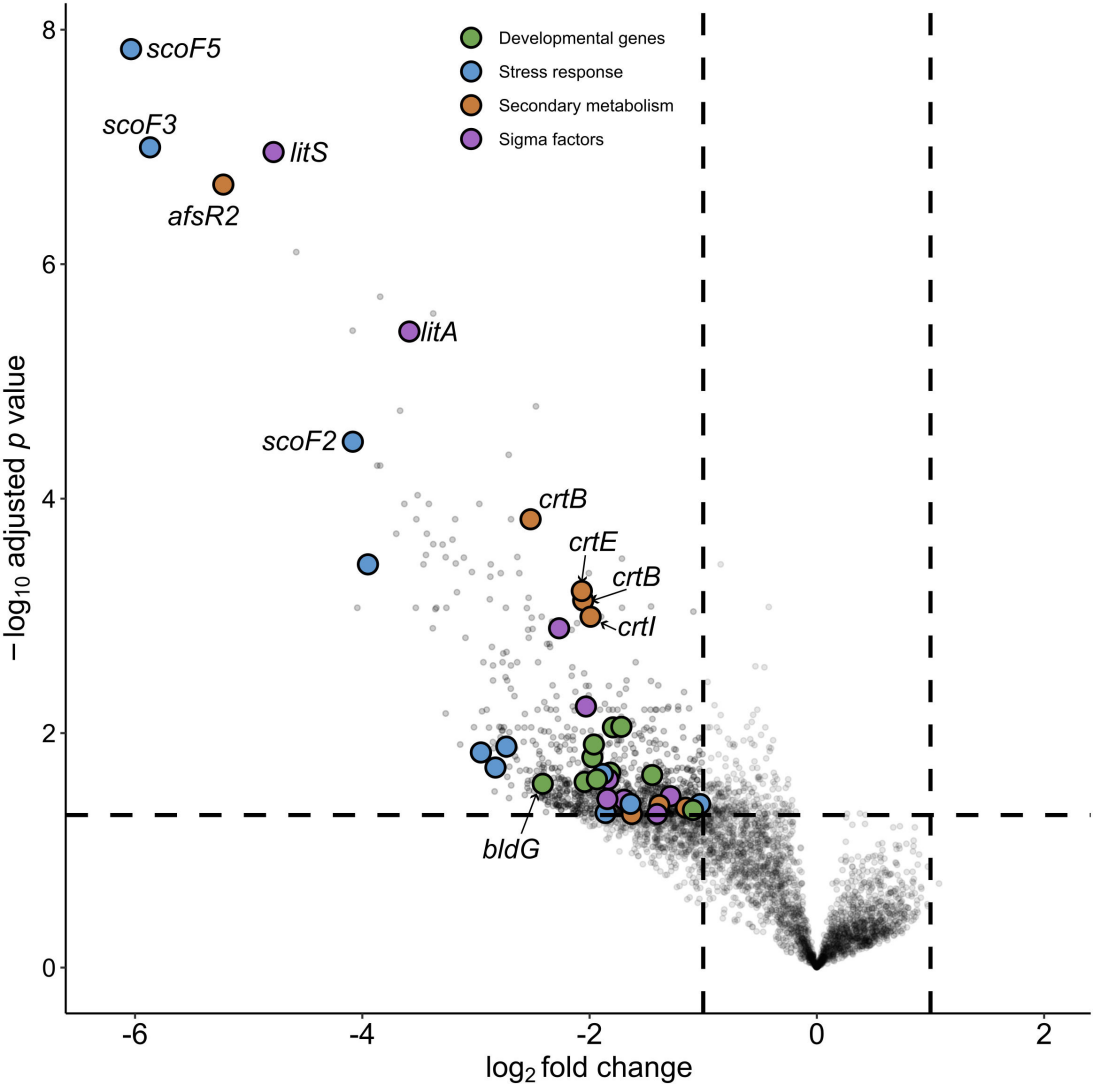
Volcano plot showing differentially expressed genes in *S. coelicolor* M1146 between day 4 monoculture vs. coculture. Genes with a negative log_2_ fold change were relatively more abundant in coculture (*n* = 4), and genes with a positive log_2_ fold change were relatively more abundant in monoculture (*n* = 4). Key genes associated with development, sigma factors, stress, and secondary metabolism were annotated using SCO identifiers via StrepDB (streptomyces.org.uk).

**Fig 7:**
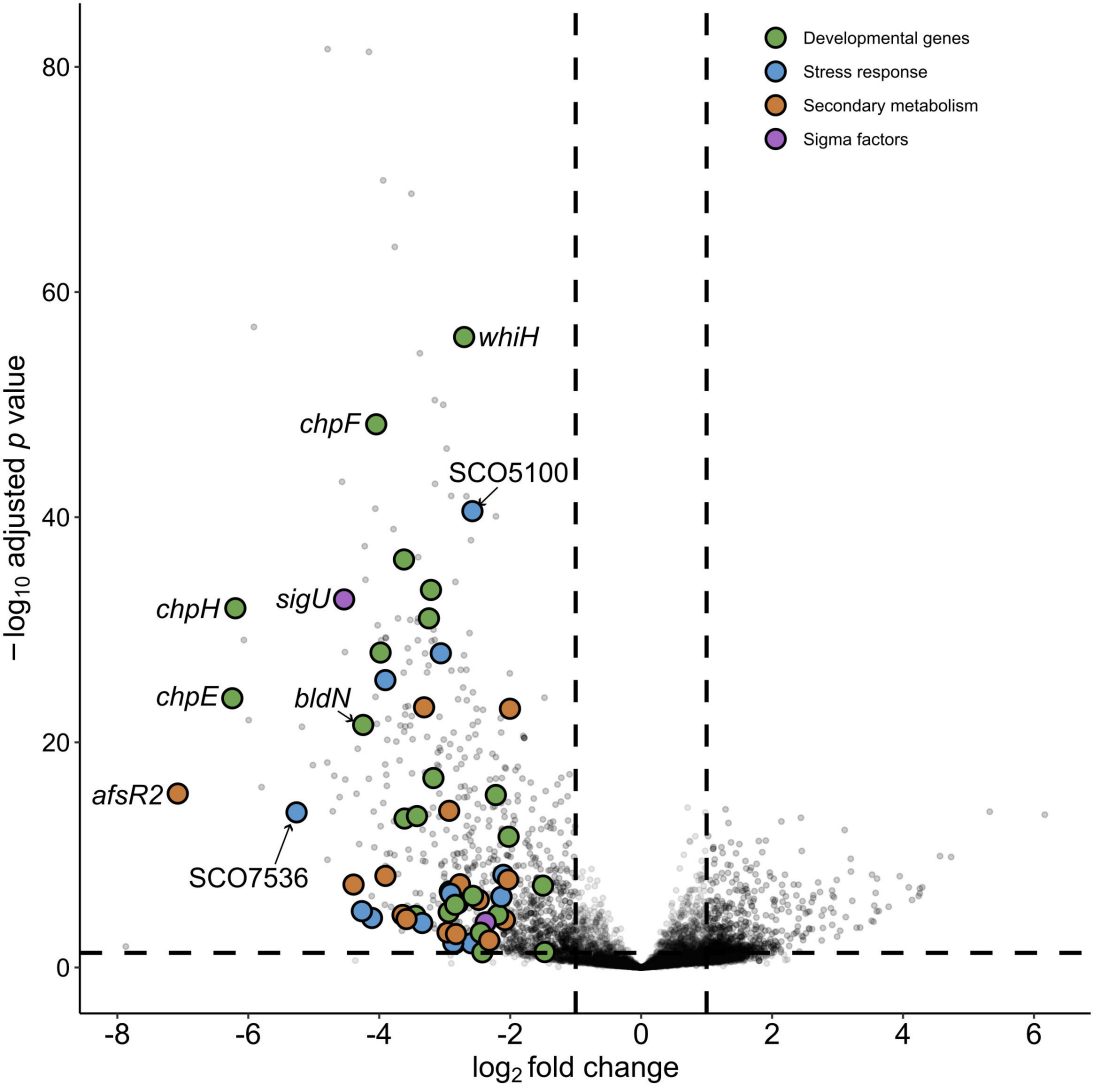
Volcano plot showing differentially expressed genes in *S. coelicolor* M1146 between day 9 monoculture vs. coculture. Genes with a negative log_2_ fold change were relatively more abundant in coculture (*n* = 4), and genes with a positive log_2_ fold change were relatively more abundant in monoculture (*n* = 4). Key genes associated with development, sigma factors, stress, and secondary metabolism were annotated using SCO identifiers via StrepDB (streptomyces.org.uk).

Morphogenesis is an irreversible phenomenon brought on in Streptomycetes by nutrient depletion (37). To probe the effect of lydicamycin exposure on morphogenesis, we evaluated the transcriptome of the receiver strain (M1146) in monoculture (MC) and coculture (CC) over four time points based on lydicamycin production (2, 4, 7, and 9 days). On day 2, there were no differential genes observed between CC and MC. Days 4 (Fig. 6) and 9 (Fig. 7) both show extensive differential expression (log_2_ and p_adj_ <0.01), 123 and 410 total genes, respectively, encompassing development, cell envelope stress, sigma factors, and secondary metabolism. Day 7 surprisingly showed very little differential expression between MC and CC. Data for days 4 and 9 can be found in the supplemental material.

Compared with *S. coelicolor* M1146 MC, *bldD* (SCO1489) and *bldG* (SCO3549) are differentially expressed in day 4 CC, and we further observe the early chaplin genes (*chpE* and *chpH*) along with *sapA* (SCO4049) differentially expressed (Fig. 6). Multiple sigma factors (*σ^H^, σ^I^, σ^N^, σ^8^*, SCO4005, and SCO3613) and regulators (*wblA*, *afsQ1/Q2*, *afsR2*, *phoR*, SCO5147, and *cvnA2/B2*), which are responsible for differentiation and antibiotic production (40), were also differentially expressed on day 4 CC (log2 fold change < −2). We also observe conservon loci (*cvnA1/B1/D1* and *cvnA13*) being differentially expressed at day 4 CC. These two loci are co-regulated with *cvn8,* which has been shown to directly stimulate antibiotic production during interspecies interactions (41).

Importantly, we observed differential expression of genes associated with various stress responses in M1146 (Fig. 5): oxidative stress response (*nur*, σ^R^, σ^8^, SCO5749, *osaB*, and *osaC*), osmotic stress (σ^H^ and σ^I^), and shock proteins (HspR, ScoF, and ScoF1-5), all of which are general responses to cell envelope stress (42). We also see differential expression of the carotenoid BGC *crt* and the neighboring sigma factor (σ^LitS^) that is both light- and stress-induced (43). BlastP on *crtY* shows a 59% homology to a putative lycopene cyclase (WJY42522.1) in *Streptomyces* sp. P9-2B2; therefore, it is logical that the yellow pigmentation in our original observation (Fig. 1A) that preceded aerial mycelium is also a carotenoid derived from this BGC.

### Lydicamycins elicit a transcriptional response like cell wall targeting antibiotics

On day 9 CC, where we observed half of the M1146 colony had transitioned to aerial hyphae, genes tightly associated with aerial hyphae formation (*bldN*, *bldM*, and *wblA*) along with hydrophobic proteins and peptides (*chpB, chpD-H,* and *sapA-B*) were differentially expressed (Fig. 7) (40). Furthermore, we see differentially expressed genes associated with kickstarting sporulation (*ssgA*, *ssgB*, *ssgR*, and s*poIIE*) and conversion of aerial hyphae to spores (*whiH*), all of which are activated by the global regulator *bldD* (log2 fold change < −2) ( 40). *wblH* (log2 fold change = −2.56), which is controlled via *whiA* (44), showed a similar transcriptional pattern to *wblA* and *bldN* and may have a similar role, but further investigations are required.

We also observe further signs of cell envelope stress on day 9 (Fig. S12). We see differential expression of a hypothetical protein (SCO5100) homologous to *ytrA* from *Bacillus subtilis*, a GntR family repressor involved in cell envelope stress response to cell wall targeting antibiotics (45). *σ^E^* is induced by a wide range of cell wall targeting antibiotics (46) and is a major marker of cell envelope stress in *S. coelicolor* (47). Along with a σ^E^-like protein (SCO4005), we observe differential expression of 12/28 tightly associated *σ^E^* regulons seen by Pospíšil et al. when *S. coelicolor* was exposed to ethanol stress (48). We also see differential expression of *σ^U^* (Fig. S7), which modulates functional proteins directly associated with counteracting cell envelope stress as well as modulating morphological differentiation (49), and several additional sigma factors (*σ*^8^, σ^F^, σ^H^, σ^I^, σ^N^, and σ^R^). Additional evidence of cell wall stress includes differential expression of SCO6091 and SCO7536, both homologs of Mycobacterial membrane protein Large (MmpL) transporters, which are pumps that send lipids to the cell envelope under stress (50). In terms of secondary metabolism, we observed differential expression of genes associated with coelibactin biosynthesis; however, it is unclear whether this is due to a generic stress response (51) or coelibactin’s ecological role in sporulation (52). We further observed differential expressions of two lanthipeptide precursor peptides (SCO6931 and SCO6932) that recently have been shown to be a part of anti-phage defense (53). Combined with the observed differential expression of a putative abortive phage infection resistance protein (FQ762_32655) that triggers cell suicide to prevent phage propagation (54), lydicamycin-induced morphogenesis or morphogenesis, in general, may trigger anti-phage defenses as part of the cell stress response.

## DISCUSSION

The ability of lydicamycin to induce sporulation places it among a select group of secondary metabolites with similar ecological roles. The only two other SMs described with sporulation-inducing effects are goadsporin and desferrioxamine E, whereas lidicamycin is more similar to goadsporin as both exhibit antibacterial and sporulation-inducing properties (9). Yamanaka et al. reported the sporulation-inducing activity of desferrioxamine E and confirmed the observation via BGC knockout studies (55). They showed their susceptible strain, *S. tanashiensis*, lacked the *des* BGC and the siderophore transporter, therefore making it prone to iron starvation. However, this is most likely a rare case amongst *Streptomycetes* as most genomes (71%) encode for the highly conserved desferrioxamine BGC (56). Additionally, several metabolites further involved in differentiation, specifically germination, have been described (37), suggesting that *Streptomyces* SMs may play larger roles in its life cycle than previously anticipated.

Two key factors likely contribute to the underreported number of sporulation-inducing SMs: (i) the low number of pathogenic *Streptomycetes* reduces the priority of biological testing in traditional drug discovery and (ii) low compound yields make it difficult to test against a broad spectrum of bacteria. The induction of morphological differentiation is a viable strategy for increasing the production of antibiotics (57, 58) and may be a route for eliciting cryptic biosynthetic pathways through subinhibitory concentrations; however, the latter requires further investigation. Thus, it is interesting to broaden ecological studies to identify metabolites capable of inducing sporulation to begin investigating the potential downstream effects. Furthermore, the investigation of ecological functions of SMs is of importance since microbes have been widely applied as biocontrol agents.

Traditional analytical techniques like HPLC or LC-MS lack the ability to quickly identify metabolites involved in cocultures unless they are produced in high titers, either lacking noticeable differences in UV chromatograms or difficulty deconvoluting many features, respectively. In contrast, MSI is a burgeoning technique equipped uniquely to handle these types of samples, as seen through this study and others (10, 59). Using MSI, we were able to quickly define potential candidate features based on their spatial distribution and through dereplication via MS and genome mining, propose, and confirm the sporulation-inducing nature of lydicamycins. However, MSI suffers from topological challenges, and actinobacteria and other filamentous microbes can be difficult to chemically image; therefore, modified strategies like what we have demonstrated or new techniques may be required to increase the utility of MSI. Furthermore, MS-based metabolomics like molecular networking and multivariate statistics continue to differentiate microbial metabolomes, as we have demonstrated with our temporal study. Although transcriptomics and proteomics have traditionally been utilized to track changes over time, metabolomics offers visibility toward the end of metabolic processes. Ultimately, all three omics provide separate details in the life of a microbe, and integration of these data will begin to reveal the larger picture of SM production.

Using MSI, we have effectively characterized lydicamycins as the responsible metabolites in a dual Streptomyces coculture through these agar-based setups. Not only did we detect the standard congeners, but we also identified several additional newly discovered and putative derivatives. Temporal production of the lydicamycins nicely correlated with self-induced sporulation of the producing strain, and the generation of a deficient mutant significantly delayed the onset of sporulation when compared to the wild type. Finally, through transcriptomics, we observed the morphogenesis transitions of M1146 alongside increasing lydicamycin exposure and observed several differentially expressed genes pertaining to cell envelope stress. This study provides an important primer for tracking down the responsible metabolites in microbial interactions but also begins to illuminate the ecological role and temporal nature of lydicamycins.

## Data Availability

LC-MS data can be found in GNPS-MassIVE at MSV000092216, and MSI data can be found at Metaspace at https://metaspace2020.eu/project/jarmusch-2023. Isolate sequencing data have been deposited at the NCBI BioProject database with accession number PRJNA985726. FBMN workflow can be found here: https://gnps.ucsd.edu/ProteoSAFe/status.jsp?task=a3dd5a7a459e475d8ca492ef7d05f3b7. Raw RNA-seq data have been deposited at NCBI SRA with accession number PRJNA1123431. All code used in this study can be found at https://figshare.com/articles/software/Files_for_the_analysis/26038807, and the output from the kallisto analysis can be found at https://figshare.com/articles/dataset/Input_files_for_DeSeq2_analysis/26029459.

## SUPPLEMENTAL MATERIAL

Data Set S1 aem.00295-25-s0001.csv Transcriptomics.

Supplemental material aem.00295-25-s0002.docx Additional methods, Table S1, and Fig. S1 to S12.

Video S1 aem.00295-25-s0003.mp4 Coculture between *Streptomyces* sp. P9-2B2 and *Kitasatospora* sp. P9-2B1 captured over a 10-day period.

Video S2 aem.00295-25-s0004.mp4 Coculture between *Streptomyces* sp. P9-2B2 and *Kitasatospora* sp. P9-2B3 captured over a 10-day period.

Video S3 aem.00295-25-s0005.mp4 Coculture between *Streptomyces* sp. P9-2B2 and *Streptomyces* sp. P9-2B4 captured over a 10-day period.

Video S4 aem.00295-25-s0006.mp4 Coculture between *Streptomyces* sp. P9-2B2 and *Streptomyces coelicolor* M145 captured over a 21-day period.

Video S5 aem.00295-25-s0007.mp4 Coculture between *Streptomyces* sp. P9-2B2 and *Streptomyces coelicolor* M1146 captured over a 10-day period.

Video S6 aem.00295-25-s0008.mp4 Coculture between lydicamycin-deficient *Streptomyces* sp. P9-2B2 and *Kitasatospora* sp. P9-2B1 captured over a 10-day period.

Video S7 aem.00295-25-s0009.mp4 Monoculture of wild type *Streptomyces* sp. P9-2B2 captured over a 10-day period.

Video S8 aem.00295-25-s0010.mp4 Monoculture of lydicamycin-deficient *Streptomyces* sp. P9-2B2 captured over a 10-day period.

ASM does not own the copyrights to Supplemental Material that may be linked to, or accessed through, an article. The authors have granted ASM a non-exclusive, world-wide license to publish the Supplemental Material files. Please contact the corresponding author directly for reuse.
